# Des Abces Phlegmoneux Intra-Pelviens

**Published:** 1845-01

**Authors:** 


### DES ABCES PHLEGMONEUX INTRAPELVIENS. Par M. *Marchal (de Calvi)*. 8vo. pp. 191. Paris, 1844. Bailliere.

THIS subject of pathological enquiry—the formation of purulent deposits within and around the Pelvis—has not, as far as we know, been made the theme of any distinct treatise in the medical literature of this country. There are many able papers or short memoirs on certain branches of the subject to be found in various surgical and obstetrical works ; but we have no summary or digested account in our language of the most important of these memoirs, nor any comprehensive description of the very serious, and, often too, very perplexing, malady which they are intended to illustrate. The French pathologists have done much more in this field of research than our countrymen ; the valuable writings of *Puzos, Levret, Raulin, Doublet, Husson* and *Dance, Dupuytren,* &c. &c. being the chief sources to which we must refer for information. Dr. *Lever*—of whose valuable paper, in a recent number of the Guy's Hospital Reports, we gave a summary in this Review for last July—deserves well of his professional brethren for having directed their attention to a class of cases that are far from being generally well understood ; and we trust that, ere long, he will again make public the results of his further experience. The present work by M. *Marchal* cannot fail to be acceptable, inasmuch as, by bringing together a large number of scattered reports, he enables the reader to judge for himself of the value of the deductions which he has drawn from them. We shall now endeavour to give as fair and faithful a review of its contents as we can, avoiding all unnecessary discussion, and confining ourselves chiefly to a condensed exposition of its most instructive contents.

As by far the greater number of cases of Intra-pelvic Abscess occurs in the puerperal state, our attention shall be chiefly directed to this form of the disease. The subject well deserves the serious study of every obstetrical practitioner; as there is often no little ambiguity in the symptoms, and it will require no ordinary tact to discriminate the true nature of the malady in its incipient stage. We trust that the following remarks may serve to throw a little light upon a somewhat obscure topic, and that even the most experienced of our readers will find, in their perusal, some suggestions that may have escaped his notice.

We shall first consider—

### The Seat of Intra-pelvic Abscesses.

Purulent matter may be formed, and collect, in various parts of the pelvis. One of the most common is the cellular tissue in the iliac fossa, between the peritoneum and surface of the *iliacus internus* and *psoas* muscles: in such cases, the abscess is said to be *sub-peritoneal.* In other cases, the pus is situated between the layers of the broad ligaments of the uterus, or in the vesico-uterine, or utero-rectal spaces; but still under or outside of the peritoneum. Occasionally it seems to be formed in the substance of the uterus itself; but, oftener, in that of one or of both of the ovaries, or, it may be, within the Fallopian tubes. Not unfrequently it is secreted from the inner or serous surface of the peritoneum, investing the womb and the adjacent organs; but, under these circumstances, the disease or lesion cannot with strict propriety be called an "abscess," *i. e.* a circumscribed deposit of purulent matter. Nevertheless, we shall afterwards find that certain purulent effusions of this nature have several points of resemblance with the subject of our present remarks, and we shall therefore briefly notice them. In a remaining set of cases, the intra-pelvic abscess is situated in, and confined to, the substance of the *iliacus internus* and *psoas* muscles, and their aponeuroses; the sub-peritoneal cellular tissue being then only secondarily or consecutively affected. We shall not at present allude to any other forms or localities of the disease; as we are unwilling to perplex the subject of our remarks by over-minute pathological subdivisions.

*Where do the abscesses usually point?* By keeping in view the few preceding remarks, the reader will find little difficulty in almost anticipating the answer to this question. When the abscess is situated in the iliac fossa, it usually points in the groin; when it occupies the substance of the psoas and iliac muscles, the pus generally works its way down to the upper and inner part of the thigh: more rarely, the abscess makes its appearance in the lumbar region. If the matter has formed in the broad ligaments of the uterus, or in any of the other intra-pelvic folds of the peritoneum, it will, in all probability, eventually burst either into the uterus or vagina, or into the bladder, or into the rectum; or, it may be, into the cavity of the peritoneum. We shall now illustrate these different terminations by cases, selected from a variety of authors. The pointing of the abscess, that is formed in the iliac fossæ, is of such frequent occurrence that our only reason for adducing an example of it is, to shew that this morbid state was not overlooked by the old authors.

*Case.*—" On the 28th of March, 1682," says Mauriceau, " I visited a woman who had been confined two months before, and in whom there had appeared a swelling in the hypogastrium, extending into the right groin. It was caused by the grosser portion of her cleansings, which, from not being sufficiently discharged, had become detained in the sides of the womb and its ligaments: these parts were much swollen, and gave rise to severe suffering. At length, an abscess was formed in the groin; it was opened by an incision, and gave issue to three cupfuls of a purulent matter that was not unlike the lees of wine. After this large swelling had continued suppurating for five weeks, the patient began to improve rapidly; ultimately she quite recovered from a disease that had put her life in great danger."

M. *Marchal* has collected together the histories of at least two dozen cases of this description, where an abscess formed in the one or the other groin of puerperal women. Several of Dr. *Lever's* cases also belong to this class.

As an illustration of the pathological anatomy of a puerperal abscess seated in the iliac fossæ, it will be useful to notice here the leading features of a case that was reported to the Anatomical Society of Paris, about ten years ago, by M. *Berard.* The woman had been seized with violent pains in the hypogastrium after her confinement. The existence of a purulent deposit in the iliac fossa had been suspected during life. On dissection, there was found a vast abscess, which commenced at the back and upper part of the left flank, between the abdominal parietes and the colon, and extended into the hypogastrium, passing between the iliac fossa and the gut; but always keeping on the outside of the peritoneum. The matter had worked its way towards the right iliac fossa—separating the peritoneum in the hypogastric region—and thence had mounted, in the direction of the median line, as high up as the umbilicus. At this part it pointed.

In the left flank, there was found a communication between the abscess and the cavity of the descending colon by a small rounded aperture.

It is difficult to determine in this case whether the intestinal ulcer was the *cause*, or the *effect*, of the enormous abscess that had gradually formed. Whatever opinion may be held on this point, let not any of our readers imagine that puerperal iliac abscesses are generally, or even often, so serious and alarmingly dangerous as was the one in the present instance. In the greater number of cases, where such an abscess points in the groin, provided there is no lesion of any internal part, we may have good hope as to the result, if the patient's constitution be moderately sound.

That an intra-pelvic abscess sometimes opens into the Vagina, will appear from the following case, that is interesting in a two-fold point of view.

*Abscess in the broad ligament, artificially opened from the vagina; also abscess in the groin; cure.*—A young woman was admitted into the Hôtel Dieu, five weeks after her confinement with her first child. A tumor had formed for some time on the left side of the hypogastrium; she had suffered from fever, vomiting, and considerable pain during the acts of defæcation and micturition. By examination *per vaginam*, a sense of fluctuation was discoverable behind, and on the right side of, the uterine orifice. M. *Recamier* made an incision upon the most prominent point of this fluctuating swelling, and gave issue to a large quantity of healthy pus. Two days subsequently, a tumor appeared in the right groin, above Poupart's ligament, and gave rise to severe pain whenever the limb was moved. Next day, fluctuation was perceived in it: the vaginal wound had already nearly cicatrised. Two small bits of caustic potash were applied to the swelling; and, on the second day after this application, an incision was made through the gangrenous integuments, and a large quantity of fætid thin pus was discharged. In the course of three weeks, the wound had closed.

M. *Bourdon*—in his valuable paper, "Des tumeurs fluctuantes du petit bassin, et de leur ouverture pratiquée par le vagin," in the Revue Medicale for 1841—has related a curious case where the fluctuation was perceptible both by the rectum and the vagina: the projection of the abscess into the gut had for some time caused a most obstinate constipation. It was opened by an incision from the vagina, and a cure was speedily effected.

MM. *Husson* and *Dance* also have described a case where the pus, that had formed in the cellular tissue of the broad ligaments, found itself a way through the substance of the cervix uteri, in which there was an irregular opening leading directly into the purulent sac.

It is to be observed that, in the cases now alluded to, the abscesses had formed external to, or *without*, the walls of the vagina; and had burst, by the usual process of ulceration, into the cavity of this canal. In other cases, however, where a gush of purulent matter has issued from the vagina, it has doubtless come down directly from the uterus; the abscess having most probably been formed in the muscular tissue itself of this organ, and eventually becoming discharged into its cavity. This indeed seems to have been the process in several of the observations recorded by our author, as well as in two or three of those narrated by Dr. *Lever*.

Several instances of intra-pelvic abscesses bursting into the *rectum* are reported by our author. His 34th case is a good example of this termination.

A young woman, about nine days after her first confinement, was exposed to cold, and became feverish in consequence. A few days subsequently, she began to complain of pain in the hypogastric and left iliac regions, in which a firm and very sensitive swelling, as big as an orange, was discoverable. By the application of leeches, and afterwards of mercurial ointment, the tenderness, but not the size, of this swelling subsided. Six weeks later, a quantity of pus was evacuated with the stools, and the patient became at once relieved from most of her former sufferings.

A somewhat similar case is reported by M. *Bouchut* from the practice of Professor *Trousseau*. It occurred in a young primiparous patient, in whom the right iliac region became the seat of a very painful phlegmonous inflammation. There was every reason to believe afterwards that pus had formed in the swelling, and had burst into the descending colon or the rectum. Madame *Boivin*, also tells us of a woman in whom, after severe pain for some time in the hypogastrium and left groin, and the appearance of a swelling there, upwards of a pint of purulent matter was one morning discharged by the rectum, in consequence of the patient's hard straining at stool.

The rupture of a pelvic abscess into the Urinary Bladder is of still more rare occurrence. The following case is adduced by our author as an instance of this lesion. It is headed thus:—

*Vast purulent collection, the consequence of accouchement, occupying the pelvis, the left iliac fossa, and the upper part of the thigh, opening spontaneously into the bladder: cure.* The swelling in the groin was at first hard and resisting; gradually it became softer, and at length a distinct fluctuation was felt in it. Much difficulty had been experienced by the patient in voiding her fæces and urine. After several days of severe suffering, she discharged a large quantity of pus with the latter excretion: this purulent admixture continued for upwards of ten days. The iliac swelling had by this time nearly quite subsided. An abscess subsequently formed at the upper and inner part of the right thigh: this was opened, and discharged a good deal of pus. Eventually the patient recovered her health, after three months' treatment.

The 48th case of our author is still more remarkable; as, there, the abscess had a double opening; one into the bladder, and the other into the rectum. It had originally formed in the right iliac fossa, and seems to have been of very large extent. After severe suffering for a considerable time, the patient began to discharge purulent matter, first with the stools, and subsequently with the urine; the size of the swelling decreasing at the same time.

In the case that immediately follows, there seems to have been an equally curious complication of lesions present: it is styled, *abscess of the left iliac fossa, the consequence of delivery, opening into the bladder and the vagina.* It is unnecessary for us to follow the particulars of the report. Suffice it to say that the patient recovered; although there still remained a good deal of swelling and tenderness in the pubic and left iliac regions, when she left the hospital. The quantity of purulent matter discharged along with the urine, and also from the vagina, had been very large.

As we might *a priori* expect, a pelvic abscess occasionally bursts into the cavity of the Abdomen: then it almost always proves fatal. In the 39th case of our author, an abscess had formed between the urinary bladder and the uterus; it opened into the abdomen, inducing a rapidly fatal peritonitis. The very next case is of a similar nature.

We have already said that purulent matter is sometimes originally secreted from, and contained within, the peritoneal lining of the pelvis;—a not unfrequent result of a partial Peritonitis after delivery. Such morbid effusions, we need scarcely add, are always of very grave import; but, let it be remembered that, even under this unfavourable state of things, the case will sometimes terminate well.

Here is a very interesting example of this lesion, recorded in the old Journal de Medecine for 1789; it is headed, *Puerperal Fever, followed by effusion into the abdomen and by an enormous deposit.*

A lady of rank became pregnant of her first child in her 24th year. The labour was tedious and difficult. On the second day she became feverish, and the hypogastrium was swollen and painful. The pyrexial symptoms subsided, but the swelling increased; and, at the beginning of the third week, there was a distinct fluctuation perceptible within the abdomen. Paracentesis was performed, and nearly six *livres* of a viscid and fætid fluid—containing numerous caseous flocculi, which sometimes obstructed the orifice of the canula—were discharged: a good deal of gas escaped at the same time. In the course of a few days, the belly became tender and swollen; and a sharp pain fixed itself in the umbilical region, where a small tumor, of the size of a nut or so, was to be felt. As this manifestly contained fluid, it was opened, and about a wine-glassful of sero-milky pus let out: there was no communication between this abscess and the cavity of the abdomen. " Four days subsequently," adds the Report, " the peritoneum suddenly opened, (at the seat of the puncture, we suppose), and a thick grayish-coloured matter, loaded with a quantity of soft flocculi, flowed out in a stream for a quarter of an hour: its odour was fætid and cadaverous. The discharge returned next day, and, again, several pounds of an offensive thick fluid escaped. This enormous deposit emptied itself, by partial evacuations, in the course of five or six days. The abdomen subsided; and all that remained was a fistula at the umbilicus, which closed several times, and gave rise to various troublesome accidents, before it finally healed: this event did not take place until the end of the sixth month. Since that time, the lady has enjoyed very passable health."

Another case is quoted from the practice of Professor *Dubois:* in this, after symptoms of metro-peritonitis, there occurred an extreme tension of the abdomen, and also a small fluctuating tumor in the groin. This was opened, and an enormous quantity of fluid was evacuated: the discharge continued for several days, and the patient gradually was restored to health. In 1763, M. *Doulcet* has recorded a case in which upwards of six pints of a fluid, having all the apparent qualities of milk, were evacuated by paracentesis from the abdomen of a woman, three weeks after her delivery. An abscess subsequently formed in the umbilical region, and discharged a milky-looking pus.

*De la Motte* has preserved the particulars of a still more singular case, in which an opening in the abdominal parietes took place spontaneously, and gave issue to a pailful of purulent matter. The patient recovered her health so completely, as to have several children afterwards.

Having now sufficiently discussed the subject of Sub-peritoneal and Intra-peritoneal puerperal abscesses, we shall proceed to notice very briefly that variety of intra-pelvic abscess, which is primarily formed in the substance or tissue of the *iliacus internus* and *psoas* muscles, and which our author designates by the appellation of *psoite puerperale.* As a well-marked illustrative example of this dangerous lesion, he has adduced the following case.

A woman, 30 years of age, was affected, soon after her tenth confinement, with severe pains in both flanks, but especially in the left one. The lower extremity on this side became much retracted, so that its extension was almost impossible. The patient was feverish during the day, had night sweats, and lost her strength rapidly. About the end of the second month after her accouchement, a tumor was perceptible in the left flank. When first examined by her medical attendant, it was soft, elastic, about as big as the closed fist, and situated equidistant between the iliac crest and the last false rib, two or three inches from the vertebral column. Anteriorly, the swelling was insensibly lost in a diffused doughy-like fullness, which extended over the whole of the left flank and iliac region. When pressure was made on the lumbar swelling, the reflux of fluid into the flank on this side was distinctly perceptible. One day, while the patient was quietly conversing with her friends, she was suddenly seized with a slight convulsive movement, and immediately expired. On dissection, not only the *psoas* and the *iliacus internus*, but also the *quadratus lumborum*, were found in a state of disorganization. There were two abscesses—the one iliac, and the other dorso-lumbar—which communicated with each other, through the *quadratus* muscle, and the anterior and middle layers of the *transversalis*. Of enormous dimensions, it was one of those purulent sacs "en forme de bouton de chemise," which M. *Velpeau* has so accurately described. The muscular debris was infiltrated with purulent matter.

In the case of M. Vigla, reported in the Bulletin of the Anatomical Society, the *psoas, iliacus internus* and *quadratus muscles* were reduced, in a great measure, to a green-coloured pulp. The sub-aponeurotic space communicated with the cavity of the cæcum by an opening through the *fascia iliaca*. There existed, at the same time, a small collection of pus in the folds of the broad ligament on the right side. The abscess extended down to the crural arch, and upwards as high as the attachments of the diaphragm. The filaments of the lumbar plexus, which were ramified on the surface, or between the superficial fibres, of the muscles, were of a dull white colour, and so soft that they were torn across with the greatest ease.

In one of the cases—described in the interesting memoir by MM. Husson and Dance, *sur quelques engorgemens inflammatoires qui se developpent dans la fosse iliaque droite*—the abscess burst through an aperture in the cervix uteri. The abscess occupied the entire extent of the iliac fossa, extending upwards to the kidney and down to the broad ligaments of the womb; the psoas and iliac muscles were dissected, so to speak, by the suppurative process, and traversed by various nervous twigs from the lumbar plexus. As the morbid action in this case commenced (it was believed) in the uterine ligaments, we cannot regard it as a genuine example of primary *subaponeurotic* abscess within the pelvis.

Besides the Suppurative Lesions which we have already considered, there is another which sometimes occurs in the puerperal state, and therefore deserves to be noticed here. In women recently delivered, the sacro-iliac symphysis sometimes becomes the seat of a purulent inflammation. The chief seat of the pain is over the point of junction of the two bones; and pressure at this part usually occasions severe suffering. "But," says our author, "the most distinctive symptom of this disease is the intolerable pain experienced when pressure is made by the finger, introduced up the vagina, in the direction of the inflamed joint: at all the other points of the vaginal circumference, pressure occasions little or no inconvenience." The patient is usually unable to walk, or even to move herself in bed, without difficulty and pain; and, ere long, the sciatic nerves are very generally involved in the morbid action, giving rise to severe neuralgic suffering along the entire course of the affected limb. *Mauriceau* has related a case, in which, after a painful and protracted labour, there was in the first instance an inflammation of the uterus, and subsequently the formation of "an immense abscess, which occupied both hips: after two months of suffering, the patient was completely cured."

Before proceeding to examine the other forms of Intra-pelvic Abscess—those, namely, which occur in the female quite unconnected with pregnancy, and also in the male subject—we shall briefly notice a few particulars, touching the history of the puerperal lesions which we have been hitherto considering, and first, as to—

*The Symptoms of their Invasion.*—The earliest symptom that is usually observed is shivering; in some cases there is only one severe fit of this; while in others the fit is frequently repeated. At the same time, the lochial discharge generally becomes much diminished or even entirely stopped, and the mammæ subside very considerably. There is always greater or less disturbance of the bowels; they are either much confined, or affected with diarrhœa. Often there is a great difficulty in micturition, and in defæcation. The movements of the hip and loins are in most cases considerably embarrassed: the corresponding lower extremity often becomes swollen and œdematous, as well as retracted or drawn up towards the body. The local symptoms will necessarily vary much according to the size of the existing swelling, its exact situation, the state of the adjacent tissues, and so forth. The judicious practitioner will never be satisfied with a mere examination of the outward parts in such cases; he will make a point of ascertaining the condition of the pelvic viscera both by the rectum and the vagina. By pressing on the abdomen with the one hand, while the forefinger of the other hand is in either of these passages, much useful information may often be acquired. The size of the tumor, when situated in the iliac region, may vary from that of a small orange to that of an infant's head or upwards. It may extend up to, or even above, the umbilicus, and round to the loin; or it may stretch down into the cavity of the pelvis, part being above and part below its brim.

*Diagnosis.*—The existence of the pelvic affection is sometimes entirely overlooked. In one of our author's cases, the physician pronounced his patient to be labouring under Phthisis, and never ascertained that there was a swelling in the groin; although the woman, who had been recently confined, complained of pain there. In another case, the disease was pronounced to be mere Neuralgia.

Again, when the presence of the pelvic tumor is known, its real nature may be quite mistaken. Thus we have known a surgeon declare an intra-pelvic abscess—which protruded at the side of the vulva—to be a cancerous growth. On the other hand, the mere accumulation of fæcal matters in some portion of the large intestine has given rise to all the usual symptoms of a purulent collection in the pelvis.

Lastly, when the existence of a fluid collection has been ascertained to be present, there may be considerable ambiguity as to its exact seat. If the contents of the swelling are great, amounting perhaps to five or six pints—as occurred in more than one of our author's cases—the medical attendant may find very considerable difficulty in deciding whether the disease be an effusion within or outside of the peritoneum, or whether it be a diffused abscess in the cellular tissue of the abdominal parietes. Professor *Marjolin* related to us, some time ago, a case where a purulent collection between the peritoneum and the abdominal muscles extended over nearly the entire surface of the belly. Under such circumstances, a false diagnosis might very readily be formed, unless the previous history of the case was perfectly well known.

After alluding to some other points in the diagnosis, M. *Marchal* concludes his remarks on this head in these words :—" In short, a woman—one, two, or three weeks after delivery—is suddenly seized with shivering and fever, perhaps without any obvious cause, or it may be after exposure to cold, or irregularity of diet, or fatigue ; she experiences a fixed pain at some one point of the abdomen; the part becomes swollen, the swelling being at first hard and resisting, and subsequently softer and perhaps fluctuating ; the corresponding thigh becomes more or less embarrassed in it movements. When such symptoms are present, we are almost authorised in predicting that we have to do with a phlegmonous tumor of the pelvis, the consequence of the puerperal state."

*Etiology.*—That there is a peculiarity of constitution in the body of a female, during the puerperal period, predisposing her to the development of purulent formations in certain parts, is a fact that is recognised by every experienced accoucheur. On this subject, our author remarks to the following effect :—

" The puerperal state is, in a great measure, explained by the condition of the blood in the parturient woman. The process of pregnancy has induced a fibrinous—if we may be allowed the expression—diathesis. The fœtus requires a constant supply of fibrine; and the female system, in consequence of this demand, acquires a singular tendency to the secretion of this formative element. But unfortunately it is, at the same time, the essential *materiel* of inflammatory action ; and thus it is that the very condition, that gives the maternal system the power of supporting the life of the young being, is the prolific source of various ills and sufferings. * * * * * *

" Thus, although the milk *en nature* is never existent in the blood, and therefore can never be transported or conveyed by *metastasis* to any local seat of inflammatory action, the elements of this secretion may be present in it at any time, just in the same manner as the elements of the fœtus are present in it during the process of fœtal development. Keeping this idea in view, we may be inclined to regard, with more favour than pathological writers in the present day are wont to do, the old theories about 'milky deposits,' 'metastatic collections,' &c. *Petit* has very happily remarked, (Dict. des Scienc. Medic.) that a woman, while suckling, has changed her very mode of existence; and we need scarcely say that the recent beautiful researches of MM. *Piorry* and *Andral* have contributed not a little to bring back the attention of pathologists to a consideration of the fluids, as well as of the solids, of the living body."

By far the most frequent exciting cause of puerperal intra-pelvic suppuration is a tedious and difficult labour. Hence the majority of cases occur after a first labour—in consequence, doubtless, of the greater degree of dilatation and contusion to which the generative organs are then successively subjected than in after-deliveries. Most of the patients, therefore, are young women, under 25 years of age. It is not improbable that the abscess is not unfrequently formed in the situation of a *thrombus*, or patch of ecchymoses, induced by the severe injury of the parts during the expulsion of the head of the child.

Some cases of intra-pelvic abscess seem to be connected with the sudden cessation of the Lochia, or of the Milk, induced by exposure to cold, by violent mental emotions, &c. " In several cases," adds our intelligent author, " the patients had left the Maternité within a few days after their delivery—a circumstance that should not be forgotten." Premature exposure is unquestionably a prolific source of disease, not only among puerperal women, but also among young infants, in France.

The induction of these abscesses appears to be sometimes—although certainly rarely—owing to a sudden suspension of the Catamenial discharge. *Andral* has related the case of a middle-aged woman, who, while menstruating, fell into a pond of water. From that time she experienced a dull, but constant, pain in the hypogastric region, accompanied with occasional vomiting and diarrhœa. She died of pneumonia about eight months after the accident. On dissection, a purulent sac was found between the uterus and rectum. On the left side of this sac, there was another tumor, which appeared to belong to the hypertrophied and degenerated ovary ; some purulent matter was found within its substance.

In not a few cases, the cause of the disease is scarcely, if at all, appreciable. We are again indebted to *Andral* for a very instructive and curious case in point. A woman, who had been long subject to protracted constipation, was distressed with a pain in the right side of the abdomen, and occasionally also with darting pain along the corresponding limb. Subsequently the chief suffering was on the other side, and was felt most in the left flank ; at which part a painful swelling made its appearance, accompanied with a distressing numbness of the corresponding thigh, which was unusually sensitive to the touch. M. *Andral* diagnosticated an affection of the ovary. The symptoms were always easier during the period that the catamenia flowed. They gradually became more alarming ; and, diarrhœa coming on, the patient died. On dissection, a purulent sac was found at the side of the uterus, and resting upon the rectum, with the cavity of which it communicated by an ulcerated opening. This purulent sac was formed by the expanded end of the Fallopian tube. Behind it was situated a smaller tumor, of the size of a chesnut: it proved to be the ovary. On making an incision into it, some healthy-looking pus flowed out. On the right side, the state of things was nearly the very reverse. There was a considerable abscess in the ovary, and not within the Fallopian tube ; although the latter was much dilated, and also contained some purulent matter.

### *Intra-pelvic Absesses in the Male Subject.*

These are sometimes the result of disease in the prostate gland, the vesiculæ seminales, or the urethra. The former organ is much more liable to tuberculous deposits, which are apt to become subsequently suppurated, than is generally imagined. Both M. *Lallemand* and M. *Ricord* have noticed this pathological fact. In some cases, the substance of the gland becomes quite melted down, if we may so speak, into purulent matter, and nothing but its investing membrane remains.

A good many cases might be adduced to shew that the formation of an abscess within some part of the pelvic basin has been attributable to, or at least connected with, Orchitis, when the spermatic cord has become severely inflamed.

*Ledran* has recorded, in his Observations de Chirurgie, 1731, a case in which the act of vomiting was followed almost immediately by a sharp pain in the right groin; next day, there was swelling and tenderness of the spermatic cord. Three weeks afterwards, a tumor had formed in the iliac region; an indistinct fluctuation was perceptible in it. After a good deal of hesitation, an incision was made into it; and forthwith a quantity of most offensive pus was evacuated. The abscess seemed to extend deep into the pelvis, following the direction of the *vas deferens*. The patient was eventually cured.

M. *Marchal* remarks, in reference to this case, "that the contraction of the abdominal muscles during vomiting powerfully closes the external inguinal ring: hence the immediate compression of the spermatic cord and its consequent inflammation. There are other efforts, besides that of vomiting, liable to produce this effect, as we have recently seen in a case under the care of M. Gerdy."

M. Velpeau, in his *Leçons Orales*, mentions the case of a young medical student, in whom there was a suppurating bubo, the result of inflammation of the testicle. In course of time, a large abscess formed in the iliac fossa, and, when opened, gave issue to a great quantity of matter. M. *Sedillot*, of the Strasbourg hospital, has recently met with two similar cases, one of which proved fatal in consequence of the supervention of hectic fever. M. *Ricord* has treated no fewer than fifteen cases of the sort: in one of these, death was induced by the dresser having pushed in some lint into the cavity of the abscess with too much force, and wounded the peritoneum.

We have alluded to the occasional occurrence of suppurative inflammation of the *iliacus* and *psoas* muscles in puerperal women. That the same lesion is sometimes met with in the male, is proved by the following cases.

A man, 40 years of age and hitherto healthy, although of a scrofulous constitution, was seized, while lifting a heavy carpet, with a sharp pain in the right loin, extending up towards the shoulder, and downwards in the direction of the small trochanter. This sharp pain was succeeded by an uneasiness in the groin of that side, embarrassing the movements of the corresponding extremity. After two months of suffering in this manner, a swelling formed in the right loin. At this time the bowels were so much confined that they did not act above once in the course of a week. On his admission into the hospital, there was a largish tumor in the iliac fossa. This gradually extended to the groin and upper part of the thigh, so that at length it occupied the entire right flank, and appeared to be continued under the Fallopian ligament. When one hand was placed on the anterior part of the swelling, and the iliac portion was tapped with the other, a very distinct fluctuation was perceptible. An incision being cautiously made through the investing tissues, until the purulent sac was reached, a large quantity of laudable matter flowed out. Upon introducing a female catheter into the wound, some days afterwards, it was found that the abscess extended backwards and outwards in the direction of the outer and posterior part of the thigh. A counter-opening was therefore deemed necessary, in order to secure a free vent for the matter. This was made a little above and behind the great trochanter: a seton was then passed between the two wounds. The amount of discharge gradually decreased, first from the anterior, and afterwards from the posterior opening: the patient eventually was restored to health.

In the following case, related by M. Fournier, the purulent deposit seems to have been owing to a *Rheumatic Cause.* A middle-aged man, after exposure to wet and cold, became affected with arthritic pains in various parts of the body: they gradually subsided under the use of anti-phlogistic remedies. There remained, however, an almost constant heavy pain in the left groin and hip; the movements of the corresponding thigh upon the pelvis being at the same time difficult and uneasy. Subsequently a swelling appeared in this groin. When admitted into the Hôtel Dieu, there was a considerable tumefaction not only in the outer part of the left iliac fossa, and in the upper and inner part of the thigh, but also over the buttock and around the great trochanter. On a careful examination of the parts, M. *Dupuytren* formed the opinion that he had to do with an immense abscess, caused by an acute rheumatic affection: he thought that, if the bones were not already diseased, the tendons and muscular aponeuroses were certainly so. An incision was made at the lowest part of the swelling behind; and a vast quantity of tolerably healthy pus discharged. The wound was closed, before the entire quantity flowed out. In the course of a few days, the matter re-collected, and was again evacuated by a puncture: on this occasion it was sanguinolent and somewhat offensive. Gaseous matter, too, had begun to collect within the sac, and was with difficulty discharged by the wound. About ten days later, a counter-opening was made in the crural region; and some dark-coloured pus, with a quantity of gas, made its escape. Irritative fever came on; and then the patient quickly sank. On *dissection,* two immense purulent sacs were found; one being situated between the *vastus externus* and the back part of the thigh: and the other on the inside of the limb, under the crural vessels, and the deep layer of the *fascia lata.* When the abdomen was opened, a purulent sac was found at the side of the sigmoid flexure: it was full of matter, gas, and detritus. Downwards, the matter readily flowed under the crural arch; and upwards, the swelling extended above the *psoas,* the substance of which, as well as of the *iliacus internus,* was in a great measure destroyed. There was no disease of the vertebræ, or of any of the bones of the pelvis.

We need scarcely say that Intra-pelvic Suppuration may be induced by various diseases and injuries of the urinary passages and the rectum, as well as by gun-shot wounds, severe contusions, surgical operations about the pelvis, and such like causes. But we have neither space nor inclination to pursue this subject at the present time.

Our remaining page we shall devote to a brief notice of those 'celebres' (as our author calls them) abscesses, which occasionally form in the right iliac fossa, in connexion with some morbid state of the Cæcum and Vermiform Appendage. This variety of intra-pelvic abscess is much more frequently observed in men than in women. Out of 36 cases, alluded to by our author, not more than four occurred in the female sex. It seems to be more common between the 20th and 30th years of life, than at any other age.

With respect to the occasional or inducing causes of the complaint, we find that, sometimes, it follows an attack of Enteritis or Entero-Colitis; and, at other times, it seems to be the result of excessive constipation, and lodgment of hard, fæculent, and other matters in the cæcum; more especially if drastic medicines have been used for a length of time.

It has been observed more than once in persons who have suffered from *colica pictonum,* and also in those who have been subject to tape-worms. But even in both these cases it is doubtful whether the antecedent maladies, or the violent remedies too often resorted to for their relief, were the immediate cause of the cæcal lesion. The presence of hard undigested substances, as melon-seeds, cherry-stones, and so-forth, has been noticed on several occasions. But, in not a few cases, it will be found difficult to trace the local mischief to any very manifest cause. Very generally, the patient's health has been ailing more or less for a length of time, before any swelling is observed in the iliac region.

An Ilio-Cæcal phlegmon has been mistaken for a tumor formed by the liver, or by the displaced kidney; for an ovarian enlargement; for a ventral hernia; for a mere accumulation of fæcal matters in the *caput cæci,* &c. The latter case mentioned is the one which is most liable to impose upon us. It is well therefore to remember that a stercoral tumor is usually irregular and knobby on the surface, and that the existing dulness on percussion may be removed by dislodging the contents of the gut. Moreover, the formation of the tumor is always preceded by constipation. Whenever there is any ambiguity in the diagnosis, we should administer aperient medicines.

As to the *termination* of the disease, we need scarcely say that, by the adoption of judicious measures in the early stage, the inflammation may often be checked before the suppurative process has commenced. When hiccup and diarrhœa ensue, or symptoms of diffused peritonitis make their appearance, the prognosis is very unfavourable. "In such cases we find on dissection," says our author, "a purulent *foyer* around and behind the cæcum, with destruction of the cellular tissue which surrounds this organ and the commencement of the ascending colon, and of that in the iliac fossa, extending down to the crural arch, or even to the pubes: the affected gut is often much softened."

Sometimes the abscess bursts into the cavity of the cæcum, either spontaneously, or after an effort of vomiting or coughing. If the fistulous opening remains, and the purulent secretion continues, the patient will, in all probability, become hectic and die; but not unfrequently the case terminates favourably; the discharge of pus becoming less and less, until at length it ceases completely. In other cases, the abscess opens into the rectum, or even into the urinary bladder. When it bursts outwardly, the quantity of purulent matter discharged has occasionally been very large: melon-seeds and other foreign substances have been known to be voided at the same time. In one of *Dupuytren's* cases, there were several fistulous openings, which gave vent to pus mixed with gaseous and fæcal matters; pus was discharged with the stools also. The patient ultimately recovered, in spite of this most unfavourable state of things. In a curious case related by M. *Meniere,* the abscess first broke outwardly; the cutaneous wound then healed, and the matter again collecting, it burst successively into the cæcum, the urinary bladder, and at length outwardly through the cicatrix in the integuments.

In all cases, the sooner that an outward opening is made with the knife, the better: by giving a free outlet to the matter as it is formed, we shall often succeed in materially lessening, if not in altogether preventing, the lesion of the gut itself.

---

### Vestiges of the Natural History of Creation.

